# Enrollment of Physicians to Continue

**Published:** 1918-09

**Authors:** Franklin Martin

**Affiliations:** Chairman, General Medical Board, Council of National Defense


### Enrollment of Physicians.

To the Editor:

1. On August 8th the following statement was authorized by the Dar Department, signed by Newton D. Baker, Secretary of War:

"TheWar Department today has suspended further volunteering and the receipt of candidates for officers' training camps from civil life. This suspension will remain in force until the legislation now pending before the Congress with regard to draft ages is disposed of and suitable regulations drawn up to cover the operation of the selective system under the new law. * * *"

Fearing that this order might be misinterpreted by doctors who would not distinguish between enlistment as a private soldier and enrollment as an officer in the Medical Reserve Corps, on August 9th I asked the Secretary of War to issue a statement making clear this point.

2. In response to this request on August 10th the following statement was authorized by the War and Navy Departments:

"Orders issued by the War and Navy Departments on August 8th suspending further volunteering and the receipt of candidates for officers' training camps from civil life do not apply to the enrollment of physicians in the Medical Reserve Corps of the Army and the Reserve Force of the Navy. It is the desire of both departments that the enrollment of physicians should continue as actively as before so that the needs of both services may be effectively met.

(Signed) JOSEPHUS DANIELS,
Secretary of the Navy.

(Signed) NEWTON D. BAKER,
Secretary of War."

3. It is desirable that the definite attention of the medical profession be called to this interpretation in order that enrollment for the Medical Reserve Corpse of the Army and the Reserve Force of the Navy which is going on so rapidly at the present time, shall not be interrupted. Trusting that you will give this prominent space in the next issue of your Journal and such editorial comment as you may deem desirable, I am,

Yours very truly,

FRANKLIN MARTIN,
Chairman, General Medical Board, Council of National Defense.

